# Effects of grassland controlled burning on symbiotic skin microbes in Neotropical amphibians

**DOI:** 10.1038/s41598-023-50394-9

**Published:** 2024-01-10

**Authors:** Laura K. Schuck, Wesley J. Neely, Shannon M. Buttimer, Camila F. Moser, Priscila C. Barth, Paulo E. Liskoski, Carolina de A. Caberlon, Victor Hugo Valiati, Alexandro M. Tozetti, C. Guilherme Becker

**Affiliations:** 1 Programa de Pós-Graduacão em Biologia, Universidade do Vale do Rio dos Sinos, São Leopoldo, RS 93022-750 Brazil; 2 Department of Biology, The Pennsylvania State University, University Park, PA 16802 USA; 3 Center for Infectious Disease Dynamics and One Health Microbiome Center, Huck Institutes of the Life Sciences, The Pennsylvania State University, University Park, PA 16802 USA; 4 Department of Biological Sciences, The University of Alabama, Tuscaloosa, AL 35487 USA; 5 Department of Biology, Texas State University, San Marcos, TX 78666 USA; 6 Programa de Pos-Graduacão em Zoologia, Universidade Federal do Pará, Belém, PA 66075-110 Brazil

**Keywords:** Fire ecology, Microbial ecology, Tropical ecology, Wetlands ecology

## Abstract

Climate change has led to an alarming increase in the frequency and severity of wildfires worldwide. While it is known that amphibians have physiological characteristics that make them highly susceptible to fire, the specific impacts of wildfires on their symbiotic skin bacterial communities (i.e., bacteriomes) and infection by the deadly chytrid fungus, *Batrachochytrium dendrobatidis*, remain poorly understood. Here, we address this research gap by evaluating the effects of fire on the amphibian skin bacteriome and the subsequent risk of chytridiomycosis. We sampled the skin bacteriome of the Neotropical species *Scinax squalirostris* and *Boana leptolineata* in fire and control plots before and after experimental burnings. Fire was linked with a marked increase in bacteriome beta dispersion, a proxy for skin microbial dysbiosis, alongside a trend of increased pathogen loads. By shedding light on the effects of fire on amphibian skin bacteriomes, this study contributes to our broader understanding of the impacts of wildfires on vulnerable vertebrate species.

## Introduction

Current climate change scenarios predict increased temperatures, more variable rainfall patterns, and more intense extreme weather events like wildfires^1^. In fire-adapted habitats, wildfires can occasionally contribute to an increase in biodiversity^2^. However, when they take place in fire-naive habitats, wildfires become a matter of critical conservation concern^3^. Controlled fire has been utilized as a preventive measure to effectively burn surplus organic matter (i.e., fuel) in natural ecosystems. The primary objective of this approach is to decrease the frequency of fire occurrences, thereby minimizing the risk of devastating wildfires^4^. Additionally, prescribed fire has been employed to manage seasonal weed growth in agricultural environments, although this particular practice is considered illegal in numerous countries^5^. Fire can dramatically impact vertebrate physiology, making animals more susceptible to diseases. Habitat loss and degradation caused by fires likely impact the immune defenses of animals. Change in host behavior and population demographics due to fires can also influence pathogen exposure and spread^6^. Nonetheless, little research has incorporated the cascading impacts of fire on wildlife health and disease risk. Considering progressing climate change scenarios and increasing agricultural expansion, fires are predicted to more frequently impact wildlife communities that are not adapted to this type of disturbance^7^.

Amphibians have received relatively little research attention in the field of fire ecology compared to other vertebrates^8^. Most amphibian species depend on permanent and temporary water bodies to complete their life cycle, are ectothermic, and have a permeable skin for osmoregulation; all of which compound their sensitivity to environmental disturbances ^9^. Fires can cause major disturbance to microhabitats by reducing humidity and increasing temperature variability^10^, and are thus likely to dramatically impact the amphibian immune system. Changes in the environmental pool of microorganisms due to fire^11,12^ could also affect the recruitment of skin-associated microbial taxa able to shield hosts from invading pathogens^13.^ Fire also induces substantial alterations in arthropod communities within the environment^14^, potentially leading to modified dietary intake patterns that subsequently diminish the ability of amphibians to combat diseases^15^.

The amphibian skin microbiome is composed of the community of symbiotic microorganisms and their metabolic products^16^ and can help regulate metabolism, influence development, and mediate immune and stress responses^17^. The microbiome also plays an important role in amphibian health through its role in defense against chytridiomycosis, a disease caused by the chytrid fungus *Batrachochytrium dendrobatidis* (Bd)^17^. This pathogen has led to widespread amphibian population declines and extinctions, including in the Neotropics^18^, but relatively little work has examined the impact of fire on amphibian host–pathogen interactions. One field study found that boreal toads from areas that have experienced natural fires have lower Bd loads, indicating that natural fires may hamper Bd’s optimal microclimates and thus could decrease the likelihood of infection^19^. Conversely, a recent observational study found that wildfires could instead increase disease risk in salamanders by altering their protective skin microbiomes^20^. Thus, controlled experimental studies are sorely needed to disentangle the links between fires, microbiome, and disease risk in wildlife.

In this study, we experimentally tested whether controlled burning alters Bd infection, and symbiotic skin bacterial composition, diversity and beta dispersion (a proxy for bacterial community stochasticity) in Neotropical amphibians sampled at a highland grassland environment over more than one year. One of our hypotheses was that controlled burning, which is commonly employed in our focal study landscape, could directly hamper the presence of Bd in the environment, consequently reducing the risk of infection in amphibian hosts. However, in the event that burning lowers the protective function of the amphibian skin bacteriome, disrupting their immune capacity, we would expect a rise in Bd infections associated with controlled burning. To examine these divergent hypotheses, we collected samples from two species of endemic treefrogs at experimental fire plots and control plots, both before and after fire treatment. Our study illuminates the consequences of controlled burning and “escaped” controlled fires on the biodiversity of fragmented agricultural landscapes, highlighting implications for the conservation of tropical amphibians.

## Methods

### Field methods

This study was carried out in an area within the lower extent of the Brazilian Atlantic Forest. The area, located in the municipality of São Francisco de Paula (29° 27′–29° 35′ S, 50° 08′–50° 15′ W), state of Rio Grande do Sul, is characterized by a mosaic of natural grasslands and subtemperate forests. The study sites are located within the Center for Research and Conservation of Nature Pró-Mata (CPCN Pró-Mata), a preserved area covering approximately 4500 ha. The local climate is classified as super-humid temperate, with rainfall well distributed throughout the year reaching 1700–2000 mm and an annual average temperature between 14 and 17 °C^21^. Although controlled burning is a regulated field management practice in the region through Law No. 11,498 of July 4, 2000, illegal burning practices have been employed frequently when carried out without authorization issued by the competent environmental agency, outside the regulations, or without limitations^22^.

**Figure 1:**
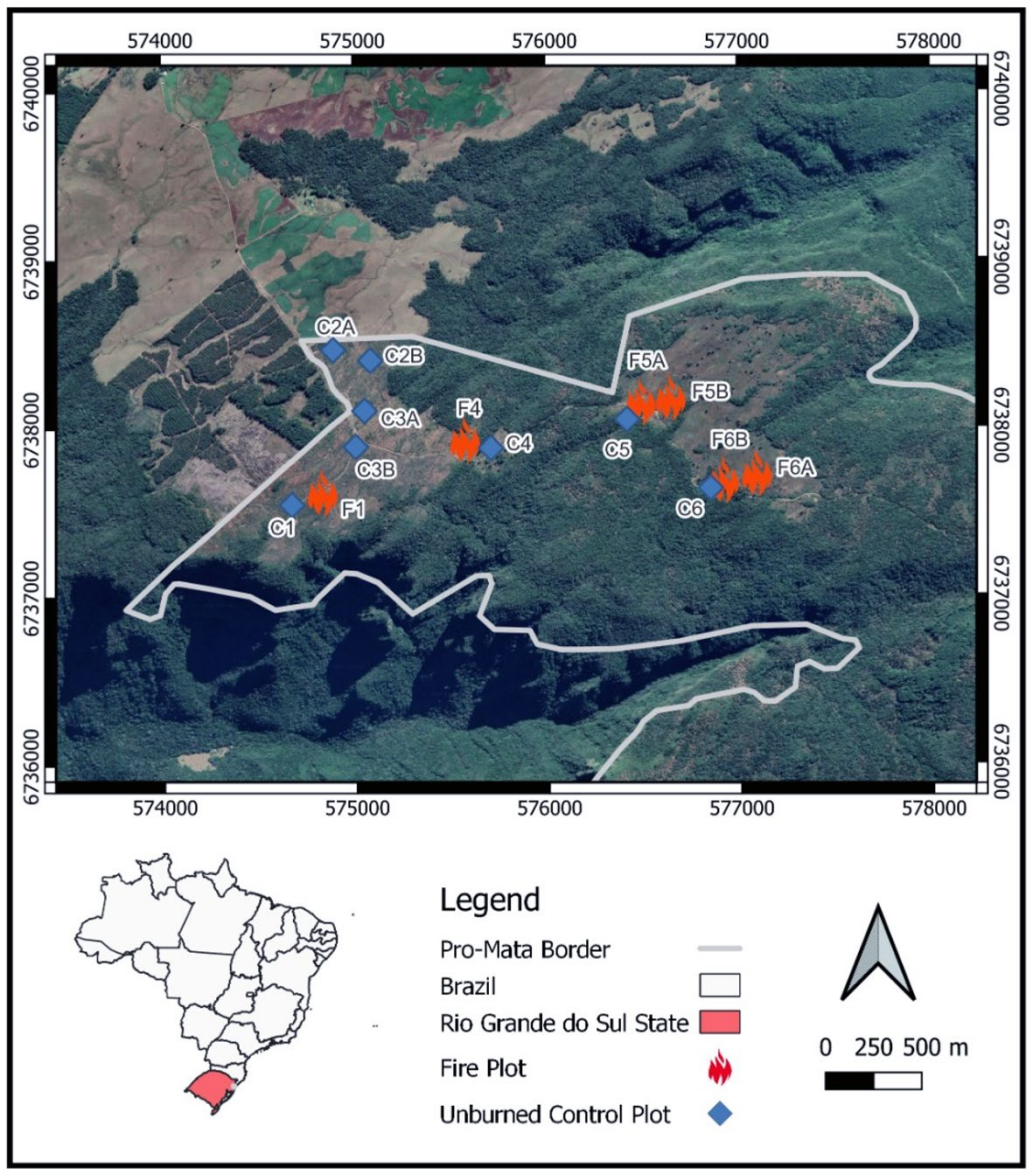
Map of sampling locations. Numbers indicate plot groups. Site IDs match those found in the supplementary data file. The map has been created using QGIS version 3.22.4 (qgis.org).

We collected skin samples from two treefrog species, the fine-lined treefrog, *Boana leptolineata* and the long-snouted treefrog, *Scinax squalirostris* (both from the Hylidae family), using rayon swabs (Medical Wire), after rinsing animals with sterile water to minimize sampling of transient microorganisms, following standard methods^23^. After sampling, swabs were stored at − 20 °C in the field, then transported to the lab on ice and stored at − 80 °C. Frogs were sampled within 14 quadrats (70 × 70 m) located in grassland areas (Fig. 1). We took advantage of an ongoing controlled burning activity that exposed six plots to a controlled burn (after our baseline sampling), while eight plots were maintained as unburned controls over the entire sampling period. Burned plots were outlined with firebreaks to keep fires contained. We carried out field campaigns September through December 2021, and January through March 2022. Burned plots 1 and 4 were burned in December 2021 and plots 5A, 5B, 6A, and 6B in February 2022 (Fig. 1). Fire plots were sampled one month after burning.

### Molecular methods

After fieldwork was completed, we extracted DNA from all skin swabs at Universidade do Vale do Rio dos Sinos, São Leopoldo, RS, Brazil using spin-column IBI extraction kits, following the standard protocol. For qPCR analysis we diluted DNA 1:10 and quantified Bd loads using Taqman qPCR assays on ITS and 5.8S genes^24^ and gBlock synthetic Bd standards diluted from 10^6^ to 10^2^ gene copies (gc). We ran plates in duplicate, with mismatching samples run on a triplicate plate. Only samples that were positive on 2 plates were recorded as positive. We averaged Bd loads across the duplicate plates and log10 transformed load values to account for non-normal residual distributions characteristic in models including pathogen load data as the response variable.

For metabarcoding bacterial communities from skin swabs, we followed the Earth Microbiome Project 16S Illumina Amplicon Protocol^25,26^, targeting the V4 region of the bacterial 16S rRNA gene using a dual-index approach with 515F and 806R barcoded primers. We PCR-amplified DNA extracted from skin swabs in duplicate plates using the following recipe per sample: 12.2 µL of UltraPure water, 4 µL of 5X Phire reaction buffer (Thermo Scientific), 0.4 µL of 2.5 mM dNTPs (Invitrogen), 0.4 µL of Phire Hot Start II DNA Polymerase (Thermo Scientific), 0.5 µL each of 10 µM barcoded forward and reverse primers (Integrated DNA Technologies), and 2 µL of sample DNA. We ran duplicate PCR plates on SimpliAmp thermal cyclers (Thermo Scientific) according to the following protocol: 98 °C for 3 min, 38 cycles of 98 °C for 5 s, 50 °C for 5 s, and 72 °C for 15 s, then 72 °C for 3 min before holding at 12 °C. We included a negative control (water without template DNA) in each plate to monitor any potential contamination of PCR reagents. We combined duplicate plates and visualized amplicons in 1% agarose gel to confirm DNA amplification, which revealed highly variable amplification among samples. We re-ran all poorly amplifying samples with doubled DNA concentration (4 µL instead of 2 µL) and halved DNA concentration (1:2 dilution) to account for low DNA concentrations and PCR inhibition. We then quantified DNA concentration for each sample using the Qubit 2.0 fluorometer with a dsDNA High-Sensitivity Assay Kit (Invitrogen) and pooled equimolar amounts of each sample (~ 10 nM) into a single amplicon library. We purified the library using a QIAquick Gel Extraction Kit (Qiagen), then measured amplicon library concentration using the Qubit 2.0 fluorometer with a dsDNA Broad-Range Assay Kit (Invitrogen). Concentrations of the purified library was 45.7 nM (11.1 ng/µL). The 16S library was sequenced using Illumina MiSeq platform using standard manufacturer protocols.

### Bioinformatics and data processing

We received demultiplexed bacterial sequences from the sequencing facility, then imported forward and reverse reads for each sample into Quantitative Insights into Microbial Ecology II (QIIME2 version 2021.11). We used QIIME2 to generate amplicon sequence variant (ASV) tables and extract metrics of alpha and beta diversity for bacterial microbiomes. Prior to analyzing sequence data, we used the DADA2 paired-end pipeline to trim sequences to 250 bp based on quality scores and cluster sequences into ASVs. We used the SILVA 138 classifier to assign taxonomy to ASVs at 99% sequence similarity. We filtered out chloroplast and mitochondrial sequences then rarefied the ASV table to 4000 reads based on rarefaction curves (Figure S1), resulting in 26 of 262 samples excluded, including all PCR controls. For analyses of alpha diversity, we calculated the ASV richness for each sample. For analyses of beta diversity, we calculated Unweighted UniFrac dissimilarity (UU) and Bray–Curtis dissimilarity (BC) between samples, and used the first principal coordinate axis (PCo1) in analysis. We calculated beta dispersion, a community stochasticity metric, that has gained recognition as a proxy for microbiome dysbiosis^27,28^ using UU and BC distance matrices partitioned by treatment, which measures the relative distance from each individual bacteriome community to the centroid of each treatment in multidimensional space (betadisper function from vegan package in program R, version 4.2.2^29,30^.

### Statistical analyses

We used Zero-inflated Negative Binomial Models (ZINB) to test the effect of experimental fire on Bd infection loads (Bd ITS counts). ZINB models provide a distinct advantage as they eliminate the need for running separate models for proportion of infected individuals and infection loads (only including Bd-positive individuals and thus reducing sample size). This consolidated approach enhances our statistical power, allowing for a more robust analysis compared to having to run two distinct models. In our ZINB model, we considered the individual and interactive effects of treatment (control or burned) and timepoint (pre- and post-fire) as fixed effects, and sampling plot as a random effect. A generalized linear mixed model (GLMM) with binomial probability distribution and logit link is also available as supplementary information.

We used generalized linear mixed models (GLMMs) with normal probability distribution and identity link function to test for the effect of experimental fire on host skin bacteriome diversity (ASV richness) and beta dispersion (UU and BC beta dispersion). In these models, we considered the individual and interactive effects of treatment (control or burned) and time point (pre- and post-fire) as fixed effects, Bd loads (log10-transformed) as a fixed effect, and sampling plot as a random effect.

Using permutational analysis of variance (PERMANOVA; adonis2 function in vegan package^29^) on UU and BC dissimilarity matrices, we tested for differences in the skin bacteriome community between treatments, timepoints, and their interaction. We conducted PERMANOVAs separately for each host species. We ran models in R version 4.2.2^30^.

Using the linear discriminant analysis effect size (LEfSe) method on the galaxy platform^31^ we tested for differentially abundant bacterial ASVs between treatments and timepoints for both host species. We ran analyses maintaining default parameters.

### Ethics declaration

All experiments were performed in accordance with relevant Federal sampling permits (SISBio #78625-1; SISGen #A7080A8). Experimental protocols were approved by Universidade do Vale do Rio dos Sinos (Unisinos) and the local animal care committee (Comissão de Ética no Uso de Animal—PPECEUA0 #02.2021). The study adhered to the ARRIVE guidelines, providing a comprehensive account of the ten essential elements necessary for describing animal research that enables reviewers to effectively evaluate the credibility our study. All animals were released at the capture location following non-invasive swabbing.

## Results

**Figure 2:**
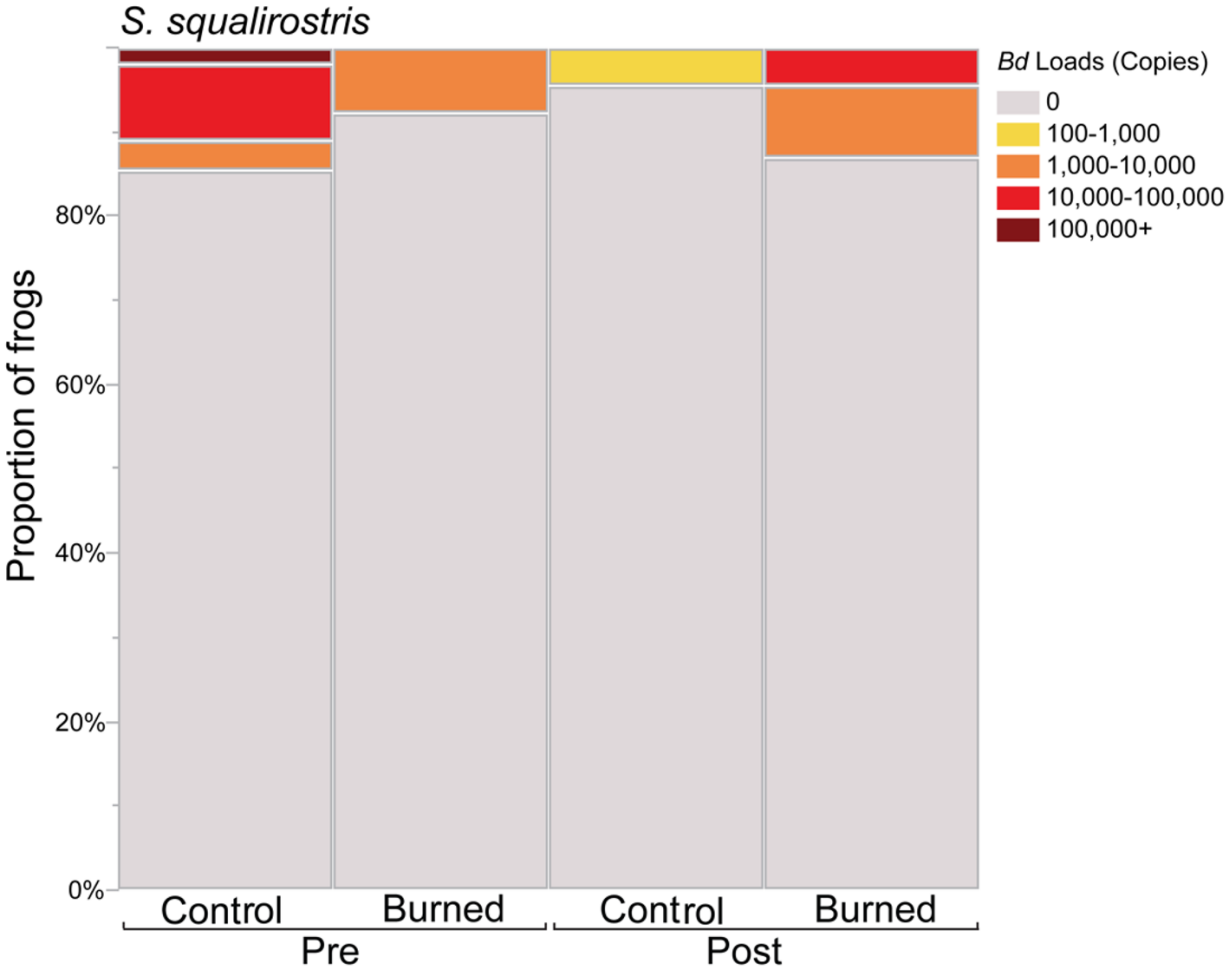
Proportions of *Scinax squalirostris* infected with *Batrachochytrium dendrobatidis* (Bd) and proportion of Bd ITS genomic copies by treatment (Control vs. burned) and time points (Pre- and Post-burning).

Overall Bd prevalence was 12.2% for *Scinax squalirostris and* 9.2% for *Boana leptolineata*. Average infection loads for the entire sampling population were 6,670 ± 46,579 for *S. squalirostris* (N = 114) and 6,970 ± 52,610 for *B. leptolineata* (N = 72). Average loads for Bd-positive individuals were 58,489 ± 130,948 for *S. squalirostris* (N = 13) and 82,489 ± 176,847 for *B. leptolineata* (N = 6)*.* Our ZINB model indicated a significant increase in Bd infection loads in individuals of *S. squalirostris* after fire plots were experimentally burned, whereas in the unburned control plots, Bd loads showed a decline trend over time (fire treatment x timepoint: Z = 3.269, P = 0.001; Fig. 2; Table S1; Table S2; Fig. S1). Despite the statistical significance of these findings, we consider these results as indicative trends due to the limited number of Bd-positive frogs in both treatments. Fire treatment had no effect on Bd loads of *B. leptolineata* (Table S1; Fig. S2), and we found no significant effect of fire treatment, timepoint, and their one-level interaction on Bd prevalence of both focal amphibian species using GLMMs (Table S3).

**Figure 3:**
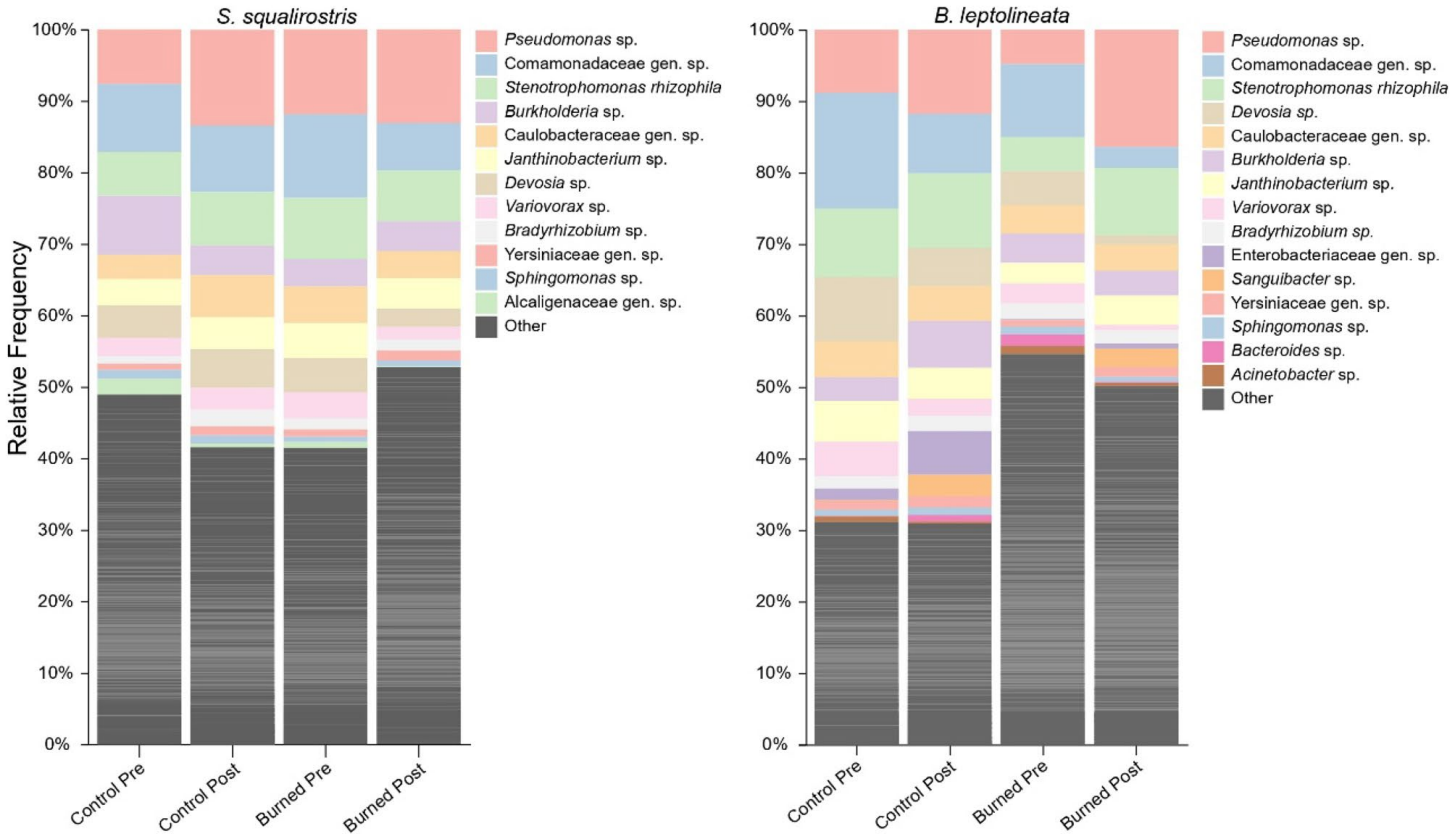
Bar plots showing frequencies of abundant bacterial ASVs across treatments and timepoints for each host species.

After filtering and rarefaction, we detected a total of 2,406 unique ASVs, with an average of 155 ± 106 from *S. squalirostris* and 198 ± 135 from *B. leptolineata*. These ASVs were primarily in the phyla Proteobacteria, Actinobacteriota, Bacteroidota, Acidobacteriota, Planctomycetota, and Firmicutes (Fig. 3). We found no significant effect of treatment, timepoint, and their one-level interaction on ASV richness for either host species (Table S5).

**Figure 4:**
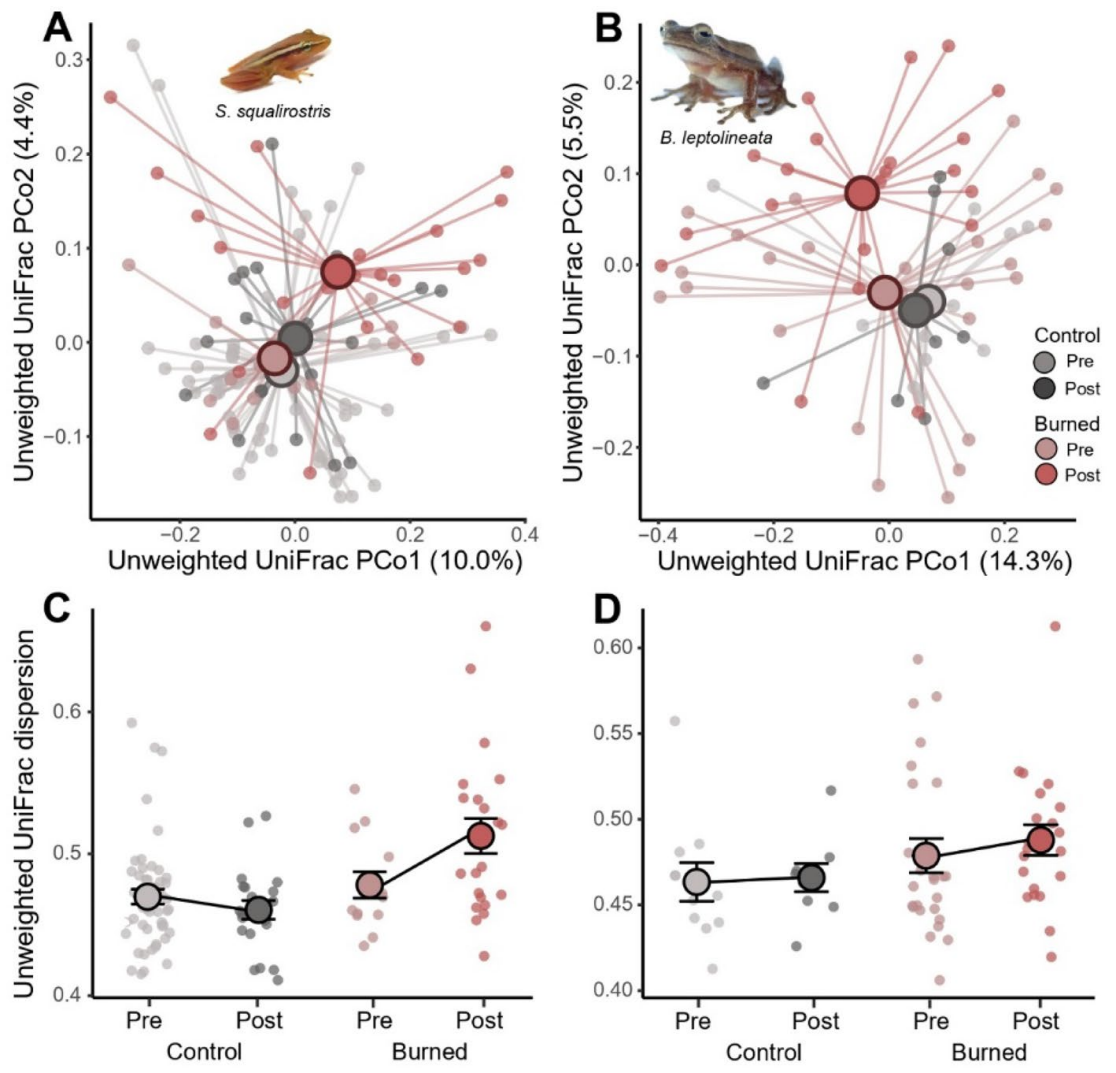
Plots showing differences in bacteriome composition between treatments. Spider plots show unweighted UniFrac skin bacteriome similarity for *Scinax squalirostris* (**A**) and *Boana leptolineata* (**B**). Centroids indicate average bacteriome composition for each timepoint within each treatment. Average dispersion between treatments over time indicate an increase in bacteriome beta dispersion, a proxy for microbiome dysbiosis, for both *B. leptolineata* (**C**) and *S. squalirostris* (**D**). Connecting lines visually highlight changes in microbiome dispersion and do not represent regression fit.

We found significant differences in skin bacteriome composition using permutational analysis of variance (PERMANOVA) on UU distance matrices between treatments, and timepoints, but not their interaction for *S. squalirostris* and *B. leptolineata* (Table S6; Fig. 4). We found similar differences in skin bacteriome composition when using BC matrices (Table S6; Fig. S3). We did not detect any differentially abundant bacteria between treatments or timepoints for either host species using LEfSe analysis.

We found a significant influence of the interaction between treatment and timepoint on UU bacteriome beta dispersion for *S. squalirostris* (Table 1; Fig. 4C). We found a similar, but non-significant trend, for *B. leptolineata* (Table S7; Fig. 4D). Finally, we found no associations between bacteriome beta dispersion and treatment, timepoint, or their interaction for *S. squalirostris* and *B. leptolineata* when using BC dispersion as response variable; (Table S8; Fig. S4 C, D).

**Table 1:** Results of generalized linear mixed models comparing unweighted UniFrac beta-dispersion of *Scinax squalirostris* bacteriomes between treatments, timepoints, and their one-level interaction.

*Scinax squalirostris*	DF	F	p
Treatment	1, 8.4	3.36	0.103
Timepoint	1, 95.2	2.95	0.089
Treatment x Timepoint	1, 94.1	4.33	0.040*
Bd Log	1, 101.7	1.59	0.211

Significant effects are indicated by an asterisk (*).

## Discussion

Throughout history, fire has exerted its influence on ecological communities and plays a pivotal role in shaping biodiversity. However, the impact of fire has expanded beyond its traditional boundaries due to human activities such as land use change and global warming. These factors have heightened the frequency and severity of fires, resulting in their unprecedented effects on biodiversity and ecosystem function^8^. In this field experiment, we found links between controlled burning, higher pathogen loads, and skin microbiome disruption in an endemic Neotropical treefrog. Specifically, we detected a marked increase in bacteriome beta dispersion and higher Bd loads in individuals of *Scinax squalirostris* after individual plots were burned.

Fire is a driver of biodiversity turnover globally, shaping communities and ecosystems ^10^. Areas that experience regular fire exhibit high levels of endemism, making fire, in conjunction with factor such as climate, resource availability, and environmental variation, a significant catalyst behind the richness of species in these regions^32,33^. Natural fire regimes creates conditions for plant species to thrive and reproduce^34^, create unique habitats where specialization can emerge^35^ and maintain a diversity of ecosystems^36^. Out of context, however, fire can have devastating impacts on ecosystems. Climate change and human-induced fires are altering fire regimes and bringing fire to places that are not fire-adapted, such as forests in South America, West-central Africa, Southeast Asia and the Tundra at the Arctic Circle^37–39^. Regions that have a long history of recurrent fire have also witnessed the occurrence of larger and more severe fires, as seen in the boreal forests of Canada and Russia^40,41^, as well as mixed forests, shrublands, and grasslands in places like Australia, southern Europe, and the western United States^42–44^. In contrast, fire-dependent ecosystems like grasslands and savannas in Brazil and the United States have experienced a irregularity or exclusion of fire activity ^45,46^. These emerging changes pose global challenges in effectively preserving biodiversity.

Fire can lead to significant changes in ecosystems, altering vegetation structure, microclimates, and microbial communities in soil and plants^11,12^. One recent review study indicated that amphibian abundance, species richness, and individual behavior are also strongly influenced by fire^47^. Specifically, 26% of the reviewed studies showed negative effects of fire, including decreased species richness^48^, 20% showed positive effects such as increased abundance ^49^, and 47% showed no significant effects^50^. Most studies focused on North American taxa were conducted in fire-dependent landscapes. In a recent study by Mulla & Hernández-Gómez^20,^ microbiome diversity of salamanders was higher in areas prone to recurrent wildfires, which could suggest colonization of opportunistic microbial taxa due to wildfires.

The combustion of vegetation and soil organic matter results in the production of ash, which can pose risks to amphibians due to its content of inorganic metals and organic polycyclic aromatic hydrocarbons. These substances are well-known for their significant toxicity, long-lasting nature, and ability to accumulate in biological systems^51^. Some studies have found that ashes can affect growth of bacteria in the skin microbiome of amphibians^52,53^. Fire-driven shifts in the skin microbiome of amphibians may in turn compromise immune system function and lead to an increased susceptibility to pathogenic infections or diseases.

Our study location is characterized by a cool, humid climate at 1000 m elevation, ideal conditions for Bd growth and transmission^54^. Despite this, we found very low Bd prevalence in our samples. We expected, according to a previous study, that fires could reduce Bd infection through shifting microenvironmental conditions beyond Bd’s optimal temperature range^19^. However, our ZINB model taking into account both shifts in baseline infection probability and Bd infection intensity pointed to a statistically significant increase in Bd loads in amphibians sampled from areas following controlled burning. While interpreting these findings with caution, our results indicate that Bd loads could still spike in amphibians through mechanisms other than suboptimal environmental conditions for Bd in post-fire environments. Our findings highlight that reduction in host immune capacity including stress and microbiome-related responses deserve further mechanistic investigation. Although we did not observe a significant link between fire and a reduction in bacteriome diversity as observed recently in salamanders^20^, the observed increase in bacteriome stochasticity is an indication that fire could indeed reduce microbiome resilience and anti-pathogen function^27^. Changes in the composition of the host microbiome after this type of environmental disturbances could also lead to sub-lethal suppression of amphibian immunity, increasing susceptibility to diseases^55^.

We found higher bacteriome beta dispersion for *S. squalirostris* in burned plots after experimental fires. Thus, our results add fire as a potential disturbance that can drive microbiome variability and, consequently, could lead to microbiome dysbiosis. Studies characterize dysbiosis as a disruption of the relationships between microbiome and host that may negatively impact host health^56^. We did not evaluate functional changes in the microbiome or aspects of host health because bacterial culturing and challenge assays with Bd were beyond the scope of our study. Although further studies are needed to unravel all the mechanisms driving the observed pattern of high microbiome stochasticity post-fire (and the Anna Karenina theory of microbiome ecology), considering beta dispersion as a proxy for dysbiosis has gained growing recognition and acceptance^27,57,58^.

Indeed, diverging microbiome community composition among individuals occupying the same environment could reflect other biological processes. For instance, reduction in habitat complexity or host movement could significantly impact exposure and microbial recruitment from divergent environmental pools, not necessarily reflecting dysbiosis. Our focal study species are commonly found foraging and vocalizing on grasses and shrubs^59,60^, which suggests that they could potentially avoid contact with soil microbial reservoirs in typical grassland conditions. After fire disturbance, we observed that grasses were completely burned, driving amphibians using those habitats to move over soil to cross the remnant vegetation (woody shrubs that were not completely burned). This change in behavior may have led to a change in the dynamics of microbial recruitment of our focal amphibian species. Additionally, burning likely causes significant changes in environmental soil chemistry and microclimates^10^, and drive nutrient runoff into amphibian breeding sites^61–63^. Even if we disregard changes in amphibian behavior, fire can alter environmental microbiomes, especially in plants and soil^11,12^. All these factors would greatly alter microbial pools in the environment and subsequently shift composition of microbial communities that are filtered/recruited into the amphibian skin microbiome, considering that most of the bacterial ASVs of amphibian skin are shared with their perching environment^13^. Although we expect that fire should homogenize the environmental microbial pool, amphibians in the post-fire treatment still showed stochastic, unpredictable bacteriome assembly compared to the control group, further supporting the Anna Karenina principle and the observed high beta dispersion as proxy for microbiome disruption.

Chronic stress driven by environmental change could also suppress host pathogen defenses^64^. Burned sites were drier than control sites due to physical drying from the fire itself and the subsequent loss of plant cover^65^. Changes in water availability in burned areas could also be an added stressor for frogs. Amphibians experiencing the stress of water loss require high energy demands and have negative cardiovascular impacts^66^. Stress hormones like corticosterone mediates the physiological response to dehydration stress^67^ and are involved in water-seeking behavior^68^. Burned areas in our study may have reduced food quality and availability^64^, disrupting corticosterone balance^69^ to levels that are potentially immunosuppressive in amphibians^70^.

This study brings novel findings linking wildfires and amphibian bacteriome health. We show that intermittent burning could have hidden effects on biodiversity through disruption of host-associated symbionts. Over time, bacteriome disruption caused by fires could potentially impact amphibian population viability, especially when combined with additional stressors like habitat loss, disease and climate change, further threatening amphibians, one of the most vulnerable vertebrate taxa.

### Supplementary Information

Supplementary Information 1.

Supplementary Information 2.

## Data Availability

Raw data used for analyses has been uploaded as a supplemental file. Any additional data is available upon request to Laura K Schuck (laurakauerschuck@gmail.com). Microbiome sequence reads generated in this study have been uploaded to the NCBI Sequence Read Archive (BioProject PRJNA999620).
