# Supplementary material for: Effects of grassland controlled burning on symbiotic skin microbes in Neotropical amphibians
Source: Sci Rep. 2024 Jan 10;14:959. doi: 10.1038/s41598-023-50394-9 (PMC10781984; doi:10.1038/s41598-023-50394-9)

**Effects of Grassland Controlled Burning on Symbiotic Skin Microbes in Neotropical Amphibians**

Laura K. Schuck^1,2,3^*, Wesley J. Neely^4^, Shannon M. Buttimer^2,3^, Camila F. Moser^5^, Priscila C. Barth^1^, Paulo E. Liskoski^1^, Carolina de A. Caberlon^1^, Victor Hugo Valiati^1^

Alexandro M. Tozetti^1^, and C. Guilherme Becker^2,3^*

**Supplementary Materials**

**Supplementary Tables**

**Table S1:** Zero Inflated Negative Binomial (ZINB) Models testing the effect of controlled fire treatments, timepoints, and their one-level interaction, and including sampling plot as a random factor, on Bd infection loads, in turn for each of the focal host species.

| **Source** | | **Estimate** | **Std Error** | **Z** | **P** |
| --- | --- | --- | --- | --- | --- |
| *Scinax squalirostris* (ZINB Model) | (Intercept) | 11.3126 | 0.4678 | 24.183 | < 2e-16 *** |
|  | Treatment | -2.9396 | 1.4022 | -2.096 | 0.03604 * |
|  | Time | -4.7339 | 1.3998 | -3.382 | 0.00072 *** |
|  | Treatment*Time | 6.7705 | 2.0713 | 3.269 | 0.00108 ** |
|  | Log (theta) | -0.5605 | 0.3451 | -1.624 | 0.10430 |
| *Scinax squalirostris* (Zero-inflation model coefficients (binomial with logit link) | (Intercept) | 1.7694 | 0.3825 | 4.625 | 3.74e-06 *** |
|  | Treatment | 0.7089 | 1.1094 | 0.639 | 0.523 |
|  | Time | 1.3038 | 1.0927 | 1.193 | 0.233 |
|  | Treatment*Time | -1.8871 | 1.6316 | -1.157 | 0.247 |
| *Boana leptolineata* (ZINB Model) | (Intercept) | 11.9456 | 0.8155 | 14.649 | <2e-16 *** |
|  | Treatment | -2.3243 | 1.2886 | -1.804 | 0.0713 |
|  | Time | -1.8976 | NaN | NaN | NaN |
|  | Treatment*Time | -0.5324 | NaN | NaN | NaN |
|  | Log (theta) | -0.6914 | 0.5194 | -1.331 | 0.1831 |
| *Boana leptolineata* (Zero-inflation model coefficients (binomial with logit link) | (Intercept) | 0.9784 | 0.6775 | 1.444 | 0.149 |
|  | Treatment | 1.5411 | 0.9998 | 1.541 | 0.123 |
|  | Time | 17.5781 | 3227.0593 | 0.005 | 0.996 |
|  | Treatment*Time | -17.0736 | 3227.0596 | -0.005 | 0.996 |

**Table S2:** Prevalence of Bd over time and treatments

|  |  | *S. squalirostris* | | | *B. leptolineata* | | |
| --- | --- | --- | --- | --- | --- | --- | --- |
| Timepoint | Treatment | Negative | Positive | Prevalence | Negative | Positive | Prevalence |
| Pre | Control | 47 | 8 | 15% | 8 | 3 | 27% |
|  | Burned | 12 | 1 | 8% | 25 | 2 | 7% |
| Post | Control | 22 | 1 | 4% | 11 | 0 | 0% |
|  | Burned | 20 | 3 | 13% | 21 | 1 | 5% |

**Table S3:** Generalized Linear Mixed Models (logistic) testing the effect of controlled fire treatments, time, and their one-level interaction, and including sampling plot as a random factor, on Bd prevalence, in turn for each of the focal host species.

| **Source** | | **Estimate** | **Std. Error** | **Z** | **P** |
| --- | --- | --- | --- | --- | --- |
| *Scinax squalirostris* | (Intercept) | -2.0082 | 0.6331 | -3.172 | 0.00151 ** |
|  | Treatment | -0.5626 | 1.2307 | -0.457 | 0.64757 |
|  | Time | -1.7146 | 1.1993 | -1.430 | 0.15281 |
|  | Treatment*Time | 2.3584 | 2.3584 | 1.345 | 0.17859 |
| *Boana leptolineata* | (Intercept) | -9.808e-01 | 6.770e-01 | -1.449 | 0.147 |
|  | Treatment | 1.545e+00 | 9.992e-01 | -1.546 | 0.122 |
|  | Time | 3.244e+01 | 5.444e+06 | 0.000 | 1.000 |
|  | Treatment*Time | 3.192e+01 | 5.444e+06 | 0.000 | 1.000 |

**Table S4:** Core bacteria for each host species. These taxa were present across 95% of samples.

| Phylum | Family | Genus (species) | Host |
| --- | --- | --- | --- |
| Proteobacteria | Xanthomonadaceae | *Stenotrophomonas* | *B. leptolineata, S. squalirostris* |
| Proteobacteria | Pseudomonadaceae | *Pseudomonas* | *B. leptolineata, S. squalirostris* |
| Proteobacteria | Oxalobacteraceae | *Janthinobacterium* | *B. leptolineata, S. squalirostris* |
| Proteobacteria | Caulobacteraceae | *uncultured* | *B. leptolineata, S. squalirostris* |
| Proteobacteria | Burkholderiaceae | *Burkholderia-Caballeronia-Paraburkholderia* | *B. leptolineata* |
| Proteobacteria | Xanthobacteraceae | *Bradyrhizobium* | *B. leptolineata, S. squalirostris* |
| Proteobacteria | Enterobacteriaceae | *Escherichia-Shigella* | *B. leptolineata, S. squalirostris* |

**Table S5:** Results of generalized linear mixed models comparing ASV richness between treatments, timepoints, and their one-level interaction for each host species.

| Source | | DF | | F | *p* |
| --- | --- | --- | --- | --- | --- |
| ***Scinax squalirostris***  R^2^ = 0.06 | Treatment | 1, | 2.0 | 1.14 | 0.400 |
|  | Time | 1, | 27.0 | 2.32 | 0.139 |
|  | Treatment*Time | 1, | 23.6 | 3.61 | 0.070 |
|  | Bd Log | 1, | 77.9 | 1.10 | 0.298 |
| ***Boana leptolineata***  R^2^ = 0.15 | Treatment | 1, | 10.4 | 0.04 | 0.848 |
|  | Timepoint | 1, | 44.9 | 0.03 | 0.855 |
|  | Treatment*Time | 1, | 49.1 | 1.24 | 0.272 |
|  | Bd Log | 1, | 58.6 | 0.34 | 0.559 |

**Table S6:** Results of PERMANOVA for analyses comparing unweighted UniFrac (A and B) and Bray-Curtis (C and D) composition between treatments and timepoints for *B. leptolineata* (A and C) and *S. squalirostris* (B and D).

|  | Response | Df | R^2^ | F | *p* |
| --- | --- | --- | --- | --- | --- |
| Unweighted UniFrac *S. squalirostris* | Treatment | 1,105 | 0.01 | 1.48 | **0.016** |
|  | Time | 1,105 | 0.01 | 1.36 | **0.044** |
|  | Treatment x Time | 1,103 | 0.01 | 1.02 | 0.398 |
| Unweighted UniFrac *B. leptolineata* | Treatment | 1,64 | 0.02 | 1.62 | **0.014** |
|  | Time | 1,64 | 0.02 | 1.47 | **0.049** |
|  | Treatment x Time | 1,62 | 0.02 | 1.12 | 0.202 |
| Bray-Curtis *S. squalirostris* | Treatment | 1,105 | 0.06 | 7.03 | **0.001** |
|  | Time | 1,105 | 0.02 | 1.81 | 0.076 |
|  | Treatment x Time | 1,103 | 0.02 | 1.70 | 0.104 |
| Bray-Curtis *B. leptolineata* | Treatment | 1,64 | 0.06 | 4.43 | **0.003** |
|  | Time | 1,64 | 0.03 | 1.92 | 0.067 |
|  | Treatment x Time | 1,62 | 0.03 | 1.80 | 0.086 |

**Table S7:** Results of generalized linear mixed models comparing unweighted UniFrac beta-dispersion of *Boana leptolineata* microbiomes between treatments, timepoints, and their one-level interaction.

| ***Boana leptolineata*** | DF | F | P |
| --- | --- | --- | --- |
| Treatment | 1, 11.3 | 0.05 | 0.823 |
| Timepoint | 1, 46.1 | 0.25 | 0.617 |
| Treatment*Timepoint | 1, 51.1 | 0.63 | 0.430 |
| Bd Log | 1, 58.9 | 0.22 | 0.640 |

Whole model test R^2^ = 0.20

- **Table S8**: Results of generalized linear mixed models comparing Bray-Curtis beta-dispersion between treatments, timepoints, and their one-level interaction for each host species.

| Source | | DF | | F | P |
| --- | --- | --- | --- | --- | --- |
| ***Boana leptolineata***  R^2^ = 0.20 | Treatment | 1, | 0.0 | 11.63 | 0.965 |
|  | Time | 1, | 1.1 | 7.84 | 0.197 |
|  | Treatment*Time | 1, | 0.3 | 0.07 | 0.886 |
|  | Bd Log | 1, | 13.8 | 0.00 | 0.955 |
| ***Scinax squalirostris***  R^2^ = 0.22 | Treatment | 1, | 7.3 | 0.36 | 0.569 |
|  | Time | 1, | 100.1 | 2.90 | 0.092 |
|  | Treatment*Time | 1, | 99.9 | 0.46 | 0.501 |
|  | Bd Log | 1, | 100.2 | 0.59 | 0.444 |

**Supplementary Figures**


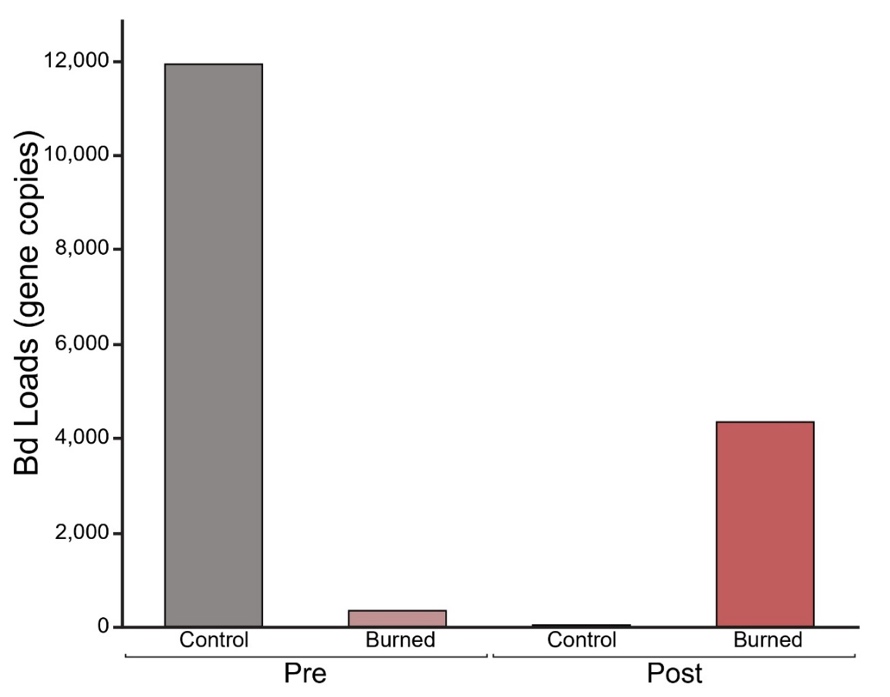
**Figure S1: Average Bd zoospore loads of *Scinax squalirostris* among treatments (Control and burned) and time points (Pre- and Post-burning). Not including zeros.**

**Figure S2: portions of *B. leptolineata* Bd loads by treatments (Control and burned) and time points (Pre- and Post-burning).**

**
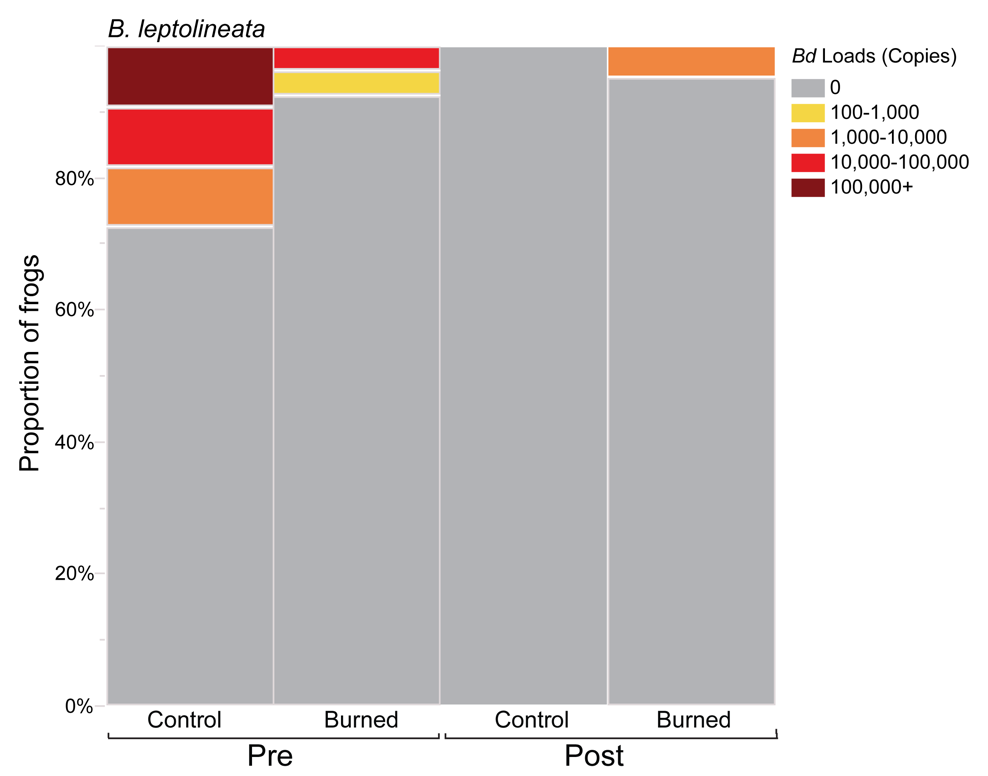
**

Figure S3: Rarefaction curves showing observed features for each sample at given sequencing depths. We rarefied at 4,000 sequence reads.


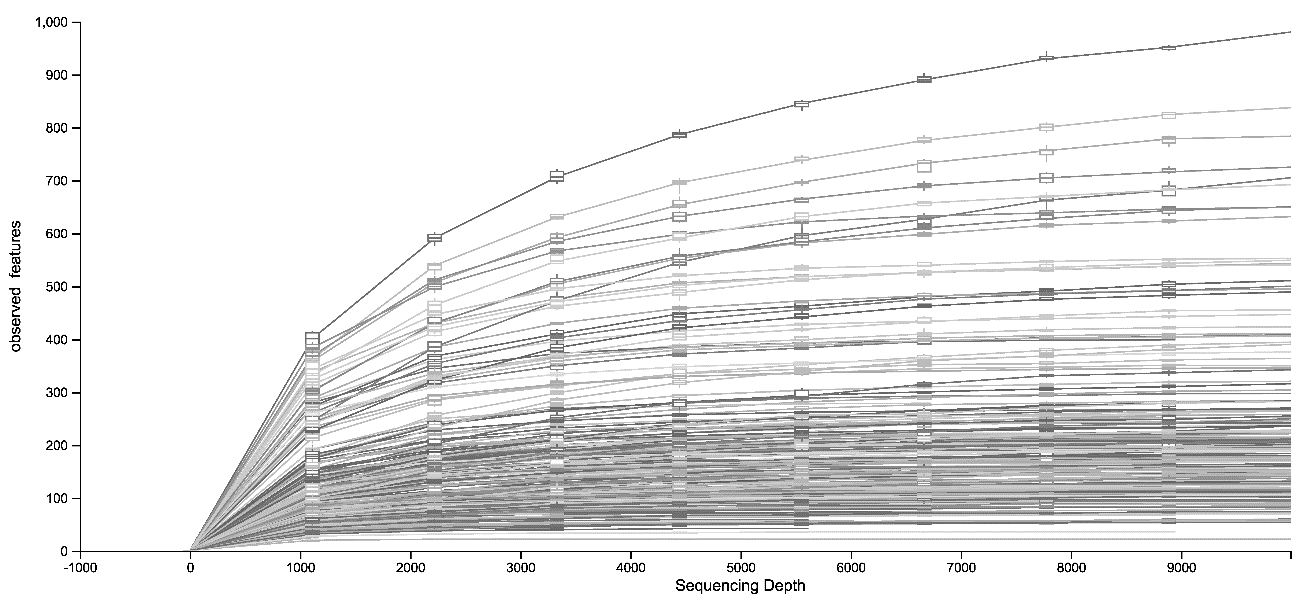


**Figure S4:** Plots showing differences in microbiome composition between treatments. Spider plots (A, B) show Bray-Curtis skin microbiome similarity for A) *Boana leptolineata* and B) *Scinax squalirostris*. Centroids indicate average microbiome composition for each timepoint within each timepoint. Average dispersion between reatments over time indicating an increase in bacteriome beta dispersion, a proxy for microbiome dysbiosis for C) *B. leptolineata* and D) *S. squalirostris*.


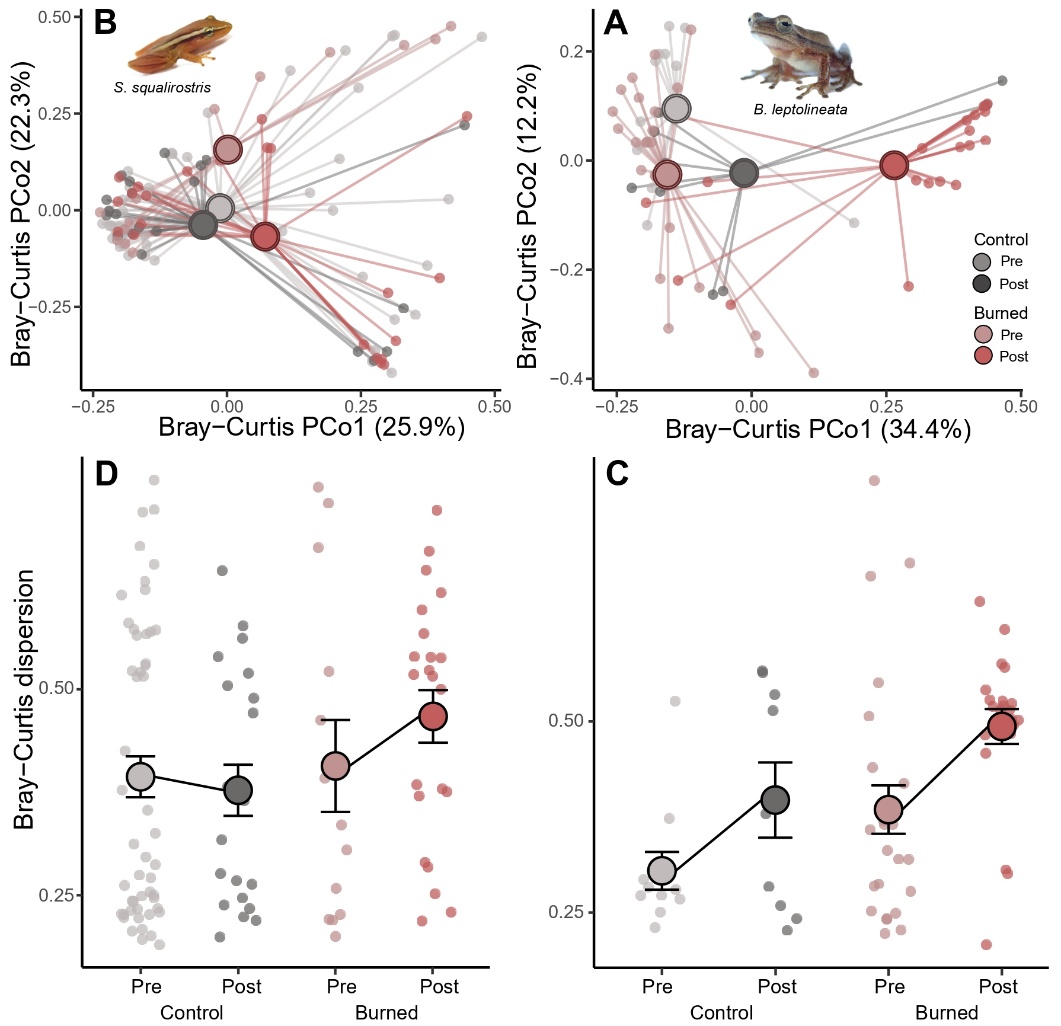

Supplement: Supplementary file 2 — Supplementary Information 2. [file 41598_2023_50394_MOESM2_ESM.docx]
